# Personal Recollections of a Dentist of the Early Days

**Published:** 1886-12

**Authors:** L. W. Bristol

**Affiliations:** Lockport, N. Y.


### PERSONAL RECOLLECTIONS OF A DENTIST OF THE EARLY DAYS.

BY DR. L. W. BRISTOL, LOCKPORT, N. Y.

READ BEFORE A UNION MEETING OF THE 5TH, 6TH, 7TH AND 8TH DISTRICT DENTAL SOCIETIES OF THE STATE OF NEW YORK, HELD IN ROCHESTER, OCTOBER 26TH AND 27TH, 1886.

At a Union Meeting of the 7th and 8th District Dental Societies, held in Buffalo, in October last, a paper was read, having for its title "Warranted."

That word has been the cause of a great deal of trouble and misunderstanding in the profession. Twenty years ago, at the bottom of every dentist's card it might be found, and in a great majority of cases the word "permanent" was added. One now scarcely can imagine how any dentist with half a grain of common sense could fail to see the absurdity of such an announcement. Yet it was the common practice in those days. We then met—as we do now—a great many unresonable people. I will give you some instances of how "warranted permanent" has served me in my practice:

I had made a partial denture on gold plate for a Mrs. S. The plate was well made and a good fit; so much so that three weeks after she said, "I like my teeth very much. If you always give as good work, I think your patients can find no fault." About two weeks after, two young ladies called on me and asked if Mrs. S. had been to see me. They were school-girls, and their school-room adjoined the room of Mrs. S. They said she had a tea cup and brush and was cleaning her teeth. She left them in a chair and went to put away the cup and brush, when one of the girls entered, took hold of the chair, and not seeing the teeth tipped them on the floor, and another girl stepped upon them and bent them. Mrs. S. was very angry, and loudly lamented her loss. The next day the old lady came to my office. She said, "Just look at my teeth; they don't fit at all, and they never did." I replied, "Why, Mrs. S., you told me last week they were a perfect fit and were satisfactory; they look as though they had been stepped upon." She replied, "No, I never told you they were a good fit; they have not been stepped upon, and you lie if you say so." She was a very zealous church member, but I handed her back the teeth, opened the door, told her to get some other dentist to do her work, for I utterly refused to do anything more for her. She replied, "You warranted them permanent, and I will sue you." I pointed to the door and told her to sue when she pleased.

I heard nothing from her for over one year and a half, when one morning a message came to me that Mrs. S. wanted to see me immediately. I had heard that she was very ill with an incurable disease. I called on her, and found her on her dying bed. She held out her hand and said she wanted to ask my forgiveness for having accused me of lying, when she was the one who lied. She had asked God to forgive her, and he had done so, and now she wanted my forgiveness. I told her that if God had forgiven her I could not do less, and gave her my hand, and left. In about two weeks she died.

I will relate another rather extraordinary case in my practice. I had made a case of teeth on gold plate for a Mr. H. They were well made and a good fit, and he was well pleased, and paid for them. About one week after, he came up behind a large, strong, athletic man and seized him around the body. The man turned suddenly and his elbow struck H. a powerful blow full in the mouth, knocking out his false teeth and loosening the teeth to which they were attached. He came to me and said, "You are not going to charge me for repairing them, are you?" I replied, "Yes; I did not warrant them against such accidents." He insisted that I should make them good, free of charge.

A day or two after I met his attorney, who said, "I am going to bring suit against you for not repairing the teeth of Mr. H." We argued the case, but he insisted that I had warranted them permanent; that it was a good accidental policy, and he would make me fix them without charge. The prospect looked fair for a law-suit and a jury trial. The lawyer was an eloquent man, a good, smooth talker. I thought I was beaten already, and I made them over. H. wore them about three weeks, when one day while leading his horse, which had a habit of throwing his head around, he was struck in the mouth, and his teeth again knocked out, breaking some of them. Here was another "warranted permanent" complication. I compromised and made them over, and got them satisfactory. In a little over four months there was a great squirrel hunt, and H. was one of the party. They had a big day of it, got back to a country tavern and imbibed rather freely, when one of the party, who was pretty full of fire water, in fun thrust the butt of his gun at H. Being a little too much intoxicated to judge of the distance, he hit him a full blow in the mouth, split open his lip, made havoc with his teeth, and "warranted permanent" came home to roost again. I made them over, adding two more teeth; in fact, made a new job of it, made out his bill, and, adding the words, "*Not* warranted for ten minutes," told him to show his lawyer the bill and receipt, and that if another accident happened to his teeth I would nail a horse-shoe over his mouth for luck. Shortly after he removed to Kansas; I never heard of him again. That attorney is not practicing in Lockport now. He has passed away, and has probably been called upon to plead in a higher court. If he escaped a verdict of guilty, I can only say that he must have pleaded earnestly.

It is now about fifteen years since I refused to administer an anæsthetic to any person unless there was a third person present, and my reason for it was this: There came to my office a lady with her daughter about seventeen or eighteen years of age. She wanted the right lower molar extracted and desired chloroform. She was seated in my chair; I prepared the napkin, poured on the chloroform and handed the bottle to her mother, telling her to place her thumb over the mouth, and if I held out the napkin to pour on a little. It was not necessary to apply more. The young lady took it kindly, and was soon under its influence. I extracted the tooth, and after a short time she came out from its influence. I asked her how she felt. She snapped out, "Don't speak to me." Her mother said, "Why do you answer the doctor so roughly?" She replied, "I did not come here to be insulted." Her mother asked her who had insulted her. She said I had; that I had hugged her and kissed her, and taken improper liberties with her. Her mother assured her that she stood right beside her every instant, and nothing of the kind had occurred. She insisted that her charge was true. I advised the mother to take her to the next room and let her lie on the lounge, and she did so. On the wall over the lounge hung a map of the village of Lockport, and on the right lower corner were the words, "Drawn by E. C. Callen, civil engineer." She caught sight of that and began to scream and cry. "There, it is drawn by Callen. I did not want it drawn by him; I wanted Bristol to draw it, and now I have been insulted and ruined." Her mother quieted her after a short time, and asked me if patients often got such whims in their heads. I told her they had all kinds of hallucinations, but in my practice I had never met with so marked a case. "Suppose," said I, "that the young lady had come here alone and gone home and told this story; what would you have thought?" She replied, "I should certainly have believed her."

I remember the case of poor Dr. Beal, of Philadelphia, who was tried, convicted and sentenced to State prison on a case precisely like this, save that I had a witness—her mother—in my favor; otherwise I might have been obliged to wear the striped clothes and look through the prison bars at Auburn. I wish to impress most emphatically upon the younger members of the profession the importance of having a third person present when giving an anæsthetic, especially to a female patient. That mother has long since gone to her grave. The daughter married, and has three children. A short time since a friend of mine, in the course of conversation, alluded to the incident, when she replied: "If it had not been for mother I always should have thought and would have taken my bible-oath that I was assaulted and ill-used at that time, and I have still the same impression."

It is now about five years since I utterly refused to administer anæsthetics and operate. I say to those applying, "Bring your doctor or call in one; there are plenty of them; let them administer it and I will operate." My reason is this :

A young lady accompanied by her brother came to have two teeth extracted, and wanted chloroform. I administered it, and extracted the teeth. She took it kindly, and came out from its influence all right, perfectly satisfied, paid her bill, and left for home. On the way the horse took fright at the cars, ran away, tipped them out in the gutter, and the lady was taken insensible to a farm house near by. They saw bloody saliva oozing from her mouth, and thought she was injured internally, but she finally recovered consciousness and told them the blood came from her mouth, for I had extracted two teeth for her. This relieved their anxiety, and they took her home. The next day one of the family to whose house she had been taken came in and told me of the accident, and said she was badly hurt. I heard nothing more for two months. In passing up the street one morning I met a lawyer, who said he wanted to see me in his office. I accompanied him, when he asked me if I remembered, two months before, giving chloroform to Miss ——, and extracting two teeth? I told him I did. "Well," said he, "she and her neighbors say she has not seen a well day since. They want me to commence a suit for damages. Cannot you and I come to a settlement without a law-suit?"

I replied, "In this case we cannot. She was not injured by chloroform. Did they not tell you that in going home from my office the horse was frightened at the cars, ran away, tipped her out in the gutter, and that she was carried in an insensible condition to a farm house, and it was a long time before they could bring her out of that condition?" "No," said he, "that is all news to me, and if it is true, we cannot make out a case." In a few days the young lady and her friends visited his office, ready to make the affidavit in a suit against me. He asked them if they had a runaway on the road home from my office, and if the young lady was pitched into the gutter and carried to a neighboring house insensible. They admitted the fact, but wanted to know what that had to do with her taking chloroform and not seeing a well day after it. He told them that if he commenced a suit against me, I could show that the injury and shock to her nervous system was what caused her illness, and that I would beat them, and they have to pay the cost of suit. He declined, under the circumstances, to bring suit, and I never heard any more of the matter.

I have said, in some of my papers, that I have spent more time working over a hot furnace trying to make continuous gum-work moulded over an impression of the mouth, than I ever shall again. Without being perfectly satisfied, or ever expecting to be, I said that John Allen had come the nearest to perfection, but his work has faults. Last August, at Niagara Falls, at the meeting of the American Dental Association, I saw some of the work done by Dr. C. H. Land, of Detroit, with his new gas-furnace, and I have seen the work in actual use, and I must now confess that in my opinion he has come nearer perfection than any one had done previously. He has overcome difficulties that with coke or coal were nearly insuperable.

The younger members of the profession cannot possibly conceive of the great disadvantages we labored under fifty years ago. Contrast those years with the present time. Look at the wonderful display of dental materials at Niagara Falls, at the convention in August; five rooms devoted to instruments, teeth and all kinds of materials required by the profession; seven different manufacturers from all parts of the United States and foreign countries. Will the coming fifty years witness as great improvements in dental surgery as the past fifty?

We live in a wonderful age of the world. In my time I have seen the Erie Canal built, the steamboat and steamships, the railroad, the telegraph, the electric light, the mowing machine, the reaper and binder, the sewing machine, and last, though not least, the stretching of a wire for hundreds of miles, which when talked to at one end the very voice is recognized at the other. I hope and pray that the coming fifty years will witness as great or even greater improvements in dentistry as in the past. I say to the young members, improve your opportunities; remember that time will plough deep furrows in your brows, whiten your hair, bring on the infirmities of age. May you then look back upon your years as well spent, the profession having been honored by your connection with it. May you then be able to exclaim, "I have filled my destiny, run my course, lived my allotted time, satisfied to step down and out, with friendship to all and ill-will to none." God bless dentistry and all who follow it conscientiously.

> "So silent time, with unresisting power,
> Labors at midnight in the lonely tower;
> Corrodes the granite in the ivied hall,
> And smiles to hear the crumbling atoms fall."

